# The 2020 Five Domains Model: Including Human–Animal Interactions in Assessments of Animal Welfare

**DOI:** 10.3390/ani10101870

**Published:** 2020-10-14

**Authors:** David J. Mellor, Ngaio J. Beausoleil, Katherine E. Littlewood, Andrew N. McLean, Paul D. McGreevy, Bidda Jones, Cristina Wilkins

**Affiliations:** 1 Animal Welfare Science and Bioethics Centre, School of Veterinary Science, Massey University, 4442 Palmerston North, New Zealand; n.j.beausoleil@massey.ac.nz (N.J.B.); k.littlewood@massey.ac.nz (K.E.L.); 2 Equitation Science International, 3 Wonderland Ave, Tuerong, VIC 3915, Australia; andrewmclean@esi-education.com; 3 Sydney School of Veterinary Science, Faculty of Science, The University of Sydney, Sydney, NSW 2006, Australia; paul.mcgreevy@sydney.edu.au (P.D.M.); bjones@rspca.org.au (B.J.); 4 RSPCA Australia, P.O. Box 265, Deakin West, ACT 2600, Australia; 5 Saddletops Pty Ltd., P.O. Box 557, Gatton, QLD 4343, Australia; cristinaluz@horsesandpeople.com.au

**Keywords:** affective state, biological functioning, behavioural interactions, human behaviour, environment, other animals, humans, welfare impacts, welfare grading

## Abstract

**Simple Summary:**

This review outlines the latest in a succession of updates of the Five Domains Model, which, at each stage, incorporated contemporary verified scientific thinking of relevance to animal welfare assessment. The current update includes, within the structure of the Model, specific guidance on how to evaluate the negative and/or positive impacts of human behaviour on animal welfare. Persons whose actions may be evaluated include, but are not limited to, livestock handlers, owners of draught animals, veterinary care staff, pound/shelter staff, zoo-keepers, wildlife managers, hunters, researchers, companion animal owners, owners of sport/recreational animals, animal trainers and service animal handlers. Situations where human–animal interactions may have negative welfare impacts include: when animals have had little or no prior human contact, when human presence adds to already threatening circumstances, when human actions are directly unpleasant, threatening and/or noxious, when humans’ prior actions are remembered as being aversive or noxious and when the actions of bonded humans cause unintended harms. In contrast, situations where human–animal interactions may have positive welfare impacts include: when the companionable presence of humans provides company and feelings of safety, when humans provide preferred foods, tactile contacts and/or training reinforcements, when humans participate in enjoyable routine activities or in engaging variable activities, when the presence of familiar humans is calming in threatening circumstances and when humans act to end periods of deprivation, inhibition or harm. The explicit delineation within the Model of the potential impacts of human interactions on the welfare of animals enhances the Model’s utility. Additional updates in this latest version are also explained.

**Abstract:**

Throughout its 25-year history, the Five Domains Model for animal welfare assessment has been regularly updated to include at each stage the latest authenticated developments in animal welfare science thinking. The domains of the most up-to-date Model described here are: 1 Nutrition, 2 Physical Environment, 3 Health, 4 Behavioural Interactions and 5 Mental State. The first four domains focus attention on factors that give rise to specific negative or positive subjective experiences (affects), which contribute to the animal’s mental state, as evaluated in Domain 5. More specifically, the first three domains focus mainly on factors that disturb or disrupt particular features of the body’s internal stability. Each disturbed or disrupted feature generates sensory inputs which are processed by the brain to form specific negative affects, and these affects are associated with behaviours that act to restore the body’s internal stability. As each such behaviour is essential for the survival of the animal, the affects associated with them are collectively referred to as “survival-critical affects”. In contrast, Domain 4, now named Behavioural Interactions, focusses on evidence of animals consciously seeking specific goals when interacting behaviourally with (1) the environment, (2) other non-human animals and (3) as a new feature of the Model outlined here, humans. The associated affects, evaluated via Domain 5, are mainly generated by brain processing of sensory inputs elicited by external stimuli. The success of the animals’ behavioural attempts to achieve their chosen goals is reflected in whether the associated affects are negative or positive. Collectively referred to as “situation-related affects”, these outcomes are understood to contribute to animals’ perceptions of their external circumstances. These observations reveal a key distinction between the way survival-critical and situation-related affects influence animals’ aligned behaviours. The former mainly reflect compelling motivations to engage in genetically embedded behavioural responses, whereas the latter mainly involve conscious behavioural choices which are the hallmarks of agency. Finally, numerous examples of human–animal interactions and their attendant affects are described, and the qualitative grading of interactions that generate negative or positive affect is also illustrated.

## 1. Introduction

The Five Domains Model for animal welfare assessment was originally formulated in 1994 [1]. It was subsequently updated in 2001 [2], 2004 [3], 2009 [4], 2012 [5], 2015 [6] and 2017 [7] to incorporate current, authenticated developments in animal welfare science thinking. The associated evolution of the Model is outlined in detail in Section 2. In general terms, the updates incorporated contemporary knowledge of interactions between physiological mechanisms and the generation of particular subjective experiences, known as affects or affective states. They also expanded the range of specific affects to be considered and clarified their biological roles. Initially, the emphasis was on welfare-compromising negative affects, and later, welfare-enhancing positive affects. Finally, the methodology for undertaking Model-based welfare assessments was refined as the Model was increasingly applied internationally to wider ranges of vertebrate species and animal use sectors.

The aim of this review is to include, within the structure of the Model, specific guidance on how to evaluate the negative and/or positive welfare impacts of human proximity to and/or behaviour towards animals. Although all published versions of the Model have included brief reference to such human impacts, usually they were portrayed simply as being potentially aversive, neutral or benign. However, during the last 5–10 years, increasing attention has been given to conducting much more detailed assessments of such impacts. The persons of interest include livestock handlers, owners of draught animals, veterinary care staff, pound/shelter staff, zoo-keepers, wildlife managers, hunters, researchers, companion animal owners, owners of sport/recreational animals, animal trainers and service animal handlers. Accordingly, the Model has been extended to facilitate explicit and detailed assessment of the welfare impacts that these people may have on the animals in their care or control.

The current review begins, as indicated above, with an account of the principal features of the ~25-year evolution of the Model (Section 2). The general features of the 2015 Model and the methodologies for grading welfare compromise and enhancement are then described (Section 3). The rest of the review focusses on the 2020 Model. Details of the first three domains (1 Nutrition, 2 Physical Environment and 3 Health), are outlined, updated and the features they have in common are identified (Section 4). We also show how a range of factors in each domain generate specific negative or positive affects that are evaluated via Domain 5, the animal’s Mental State. Domain 4 and its attendant Domain 5 affects are then described in detail (Section 5). Previously called “Behaviour” and now “Behavioural Interactions”, Domain 4 is subdivided according to the nature of animals’ interactions with (1) their environment, (2) other non-human animals and (3) humans. The last of these is described extensively, including consideration of the grading of negative and positive welfare impacts. Finally, the review ends with concluding comments (Section 6).

## 2. The 25-Year History of the Five Domains Model: Responses to Changes in Animal Welfare Thinking

### 2.1. Formulation of the Model for Assessing Negative Impacts of Research, Teaching and Testing

The Model, originally formulated in 1994, had the specific purpose of prospectively and retrospectively assessing and grading the negative impacts of research, teaching and testing (RTT) procedures on sentient animals [1]. Deployment of the Model enabled such assessments to be made in much greater detail than before [1,3,8,9,10]. In 1997, assessments and grading using the Model were mandated within the regulations that govern animal ethics committee scrutiny of all proposed and completed RTT activities in New Zealand [10], a requirement that continues to this day.

Prior to formulation of the Model, RTT impact assessments usually focussed very narrowly on the precise details of the particular manipulation(s) to be applied to the animals, leaving largely unexamined the animals’ wider circumstances that could cause additional negative impacts [1,8,9,10]. The first four of the five domains of the Model were developed to correct this [1]. Their purpose was to draw attention both to interactions among diverse functions within the body and to the negative impact of external factors on those functions, all of which, in various combinations, have relevance to impact assessments across the four physical/functional domains. Finally, the fifth domain was designed to capture the overall mental experience of the animals, evaluated in terms of the suffering from all impacts considered within the first four domains [1]. Hence, the explicit focus of the 1994 Model was the detailed and holistic assessment of animal welfare *compromise*. It also provided a basis for qualitatively grading the severity of the negative impacts [1,3].

**Figure 1:**
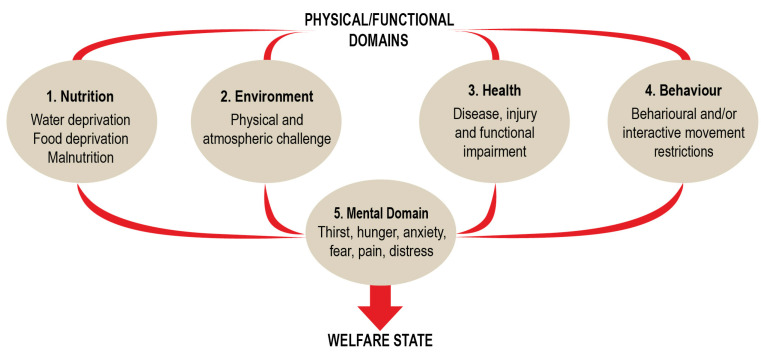
The 1994 Five Domains Model, redrawn from Reference [1].

The five domains were: (1) nutrition, (2) environment, (3) health, (4) behaviour and (5) mental state. The first three domains focussed attention on *internal* imbalances or disturbances which had nutritional, environmental and health origins. In contrast, the focus of the fourth domain was on *external* restrictive confinement or restraint, or otherwise unusual space availability and/or negative impacts of the presence or absence of other animals (including humans) [1,3]. After collation of the objective evidence derived from consideration of factors in the first four domains, the subjective, emotional or affective experiences, cautiously inferred to be associated with these disturbances or restrictions, were then assigned to the fifth domain [1,3,4]. The fifth domain enables an ultimate assessment of the overall welfare state of the animals, understood in terms of what they were likely to experience subjectively (Figure 1). Notably, the first version of the Model restricted these experiences to thirst, hunger, anxiety, fear, pain and, as a catchall term, ‘distress’ (Figure 1) [1].

From the outset, the Model was based on the premise that physiological mechanisms, later generalised in the term “biological functioning” [11], are the foundation of affective experiences, that affective experiences can influence physiological mechanisms and that both of these elements interact dynamically within the body which operates as an integrated whole entity [1]. However, around this time, two competing schools of thought emerged, one emphasising “biological functioning” and the other “affective state”, each of them arguing that the other had significant shortcomings in the ways it assessed animal welfare (see References [7,11,12]). Now, it is widely recognised that these two elements interact dynamically and that together they provide a more comprehensive foundation upon which to base welfare assessments [7,13,14,15,16,17]. Also, the inclusion of Domain 5, mental state, within the Model emphasises that what matters to animals in welfare terms is their subjective experiences. The 1994 Model therefore had an affective state orientation, but with the advantage, carried forward into all later versions of the Model, that the dynamically integrated alignment of the physiological mechanisms underlying specific affects provided a more coherent and informative basis for evaluating their welfare significance.

### 2.2. The Initial Emphasis on Negative Welfare States

The inception of animal welfare science occurred when the scientific method was first applied to evaluating problems perceived to have welfare significance [13]; for example, those in production animals exposed to inadequate nutrition, shelter/shade/space and protection against disease and injury [4,18,19,20]. From the outset, animal welfare science focussed on the optimal care of animals’ physical/functional states, the aim being to be free of any identified problems [4,12,16,18,19,21]. In animal welfare terms, this meant that, for about 15 years, virtually all scientific attention was focussed on studying negative welfare states and the circumstances that caused animals to have unpleasant or aversive experiences [4]. The impacts of this approach were profound. It resulted in major science-based advances in understanding of animal welfare and its management (e.g., References [4,13,18,19,20,22,23,24,25,26,27,28,29]), advances which provided the foundations for the subsequent developments in thinking and ways of assessing welfare states, some of which are described below.

### 2.3. Giving Greater Definition to the Meaning of “Distress”

As noted above, the fifth domain of the 1994 Model drew attention to a limited range of specific affective experiences and, as a catchall term, to “distress” (Figure 1) [1]. This was deliberate because the inclusion of additional specific affects was thought likely to hinder acceptance of the Model at a time when the legitimacy of focusing on affective states had not yet been widely accepted among animal welfare and other animal-based scientists [11,13,14]. So “distress” became a “place-holder” for other negative affects animals may experience.

Use of this term, and equally generic references to “suffering”, are still common today in animal welfare legislation, codes of welfare and legally enforceable regulations, and are also included in industry and institutional guidelines [30,31]. Nevertheless, it was increasingly recognised that such generic terminology can oversimplify the way animal welfare is formally and informally evaluated and regulated (see below). Accordingly, over at least the last 20 years, considerable attention has been given to identifying specific affects that may be included in these terms [6,16,25,31,32,33,34]. Thus, the list expanded, and in addition, two major categories of negative affect were identified [6].

The first category, survival-critical negative affects, refers to experiences generated by sensory inputs that register imbalances or disruptions in the *internal* physical/functional state of animals. They include breathlessness, thirst, hunger, pain (~30 varieties), nausea, dizziness, debility, weakness and sickness [4,6,23,32,35,36,37,38,39]. These affects are designated as survival-critical because they are aligned with essential components of genetically embedded mechanisms that elicit or are associated with behaviours on which the survival of the animals depends [35,37,38].

The undoubted negativity of each affect creates a sense of urgency, or a dominating compulsion, to engage in behaviours which are specific to that affect and its resolution (e.g., References [35,38]). Examples of links between affects and responses include breathlessness and respiratory activity, thirst and water seeking/drinking, hunger and food acquisition, pain and escape or avoidance responses to injury, as well as weakness/sickness and securing benefits from isolation and rest [4,6,23,32,36,38,40,41]. Importantly, the greater the intensity of the negative affect, the greater the sense of urgency or compulsion to engage in the aligned behaviour, and vice versa. Once the behaviour achieves the required corrective physical/functional outcome, the intensity of the negative affect declines and, correspondingly, the motivation to perform the salient behaviour subsides [35,38]. Unpleasant experiences that cannot be effectively relieved through behavioural and physiological responses may have a greater detrimental impact on the welfare state than acute but short-lived experiences.

The second category, situation-related negative affects, refers to experiences generated by brain processing of sensory inputs that mainly originate from outside the body and reflect the animal’s perception of its *external* circumstances, i.e., its situation [16,38]. These affects currently include frustration, anger, helplessness, loneliness, boredom, depression, anxiety, fear, panic and hypervigilance (see References [7,16,25,37,42,43,44,45,46,47,48,49,50,51,52,53]). Also note that the emotional pain of social isolation, i.e., loneliness, is now receiving increasing attention [52,54]. Animals in impoverished and/or threatening situations may experience these affects in various combinations.

The distinguishing attributes of each negative affect in these two categories have now been described [55]. Identifying the specific conditions that generate this wide range of negative affects and understanding the bases of their two categories, allows potential negative welfare impacts to be assessed more thoroughly and remedial actions to be focussed more precisely than before [6,16,31]. It is worth noting that the two categories are not mutually exclusive. For example, a tired racehorse that is being whipped may feel pain triggering escape or avoidance responses, and helplessness if those responses do not resolve the situation because the horse cannot escape the jockey who is the source of the pain [47,48]. Likewise, the experience of pain may be modulated by awareness of fear-inducing stimuli such as the presence of predators [56].

### 2.4. Including Consideration of Positive Affective Experiences in the Model

From the early 2000s, animal welfare scientists gave increasing attention to positive affective experiences (for References see [5,13,14,16,21,27,35,37,39,45,51,54,57,58,59,60,61,62,63]). This was motivated by the recognition that good or acceptable animal welfare, embodied in notions such as “a life worth living” [14,16,21,57], cannot be achieved simply by mitigating or avoiding negative experiences and that some pleasurable experiences are needed as well. Thus, attention increasingly shifted away from the mere care of animals towards their psychological well-being. The Model was therefore revised extensively to include, in each of the first four domains, the internal and external circumstances that may give rise to positive affects which, as with the negative affects referred to above, were assigned to the fifth (mental) domain for consideration [6]. Such experiences, when present, contribute to welfare enhancement.

These revisions were based on the scientifically supported understanding that animals may have pleasurable experiences when their external circumstances include, but are not limited to, the following: variability that provides an optimal balance between predictability/controllability and novelty/unpredictability, meeting species-specific needs for movement and exercise, access to preferred sites for resting, thermal comfort and elimination behaviours, environmental choices that encourage exploratory and foraging behaviours and durations, availability of a variety of feeds having attractive smells, tastes and textures, and circumstances that enable social species to engage as fully as possible in bonding activities with familiar conspecifics, the calming comfort of being in a group of familiar conspecifics and, as appropriate, other affiliative interactions such as allogrooming, bonding, maternal, paternal or group care of young, play behaviour and sexual activity [6,7,16,21,51,54,59,60] (see Section 5). Expressed in general terms, the associated welfare enhancing affects likely include various forms of comfort, pleasure, interest, attachment, confidence and a sense of being in control (see Section 5.2 on agency) [7,13,16,21,27,58,63,64].

### 2.5. Applying the Model to Numerous Species of Sentient Animals Evaluated for Diverse Purposes

Within the mandatory New Zealand regulatory context, the Model in its various updated versions (e.g., References [2,3,4,5,6,7,39]) has been used to assess the negative welfare impacts of RTT procedures applied to a wide range of sentient animals being evaluated for diverse purposes. As noted previously [12], the animals have included horses, cattle, deer, goats, sheep, pigs, poultry, game birds, other birds including endemic, native and introduced species, dogs, cats, guinea-pigs, mice, rats, rabbits, ferrets, stoats, weasels, kangaroos, wallabies, possums, cetaceans, reptiles, amphibians and fish. The studies’ purposes have included fundamental and applied biomedical, veterinary, agricultural, ecological, welfare, educational and other approved investigations [12].

### 2.6. Expanding Application of the Model Beyond the Research, Teaching and Testing Context

In addition to these RTT purposes, the Model has also been used to prospectively and/or retrospectively assess negative and/or positive welfare impacts of proposed new or modified approaches to housing, managing and/or interacting with farm [4], working [65], livestock guarding [66], sport [67,68,69], zoo [4,70,71,72,73,74], wild [75], free roaming [76], introduced [56,77,78,79,80,81,82] and other terrestrial animals [4], as well as cetaceans [83,84]. The Model has also been used forensically in Canadian court cases to assess suffering and animal cruelty [55].

Given the diversity of animals and Model applications, there is merit in assembling scientifically informed experts who collectively can provide detailed input on species-specific biology, ethology, ecology, physiology, pathophysiology, health and management (e.g., Reference [85]), and also, affect-related, neuroscience-supported behavioural expertise, and experience with the operation of the Model [6,7,16,32,76,81]. Using widely experienced panels or consultative networks is helpful in such evaluations (e.g., References [58,66,67,70,78,80,82,84,86]).

## 3. The 2015 Five Domains Model

Full descriptions of the 2015 Model, including details of how it operates and its key applications to the assessment and management of animal welfare, have been published elsewhere [6,7,16,55,65]. It is strongly recommended that readers consult these sources after perusing the brief outline provided below.

### 3.1. General Features of the Model

The Model is not intended to define good and bad welfare, nor is it intended to accurately depict body structure and function. Rather, it is a device for facilitating systematic, structured, thorough and coherent assessments of animal welfare, and for qualitatively grading welfare compromise and enhancement (see Section 3.3) [6,7,16]. The purpose of each domain is to draw attention to areas that are relevant to welfare assessments, taking into consideration the understanding of animal welfare briefly outlined above and presented in more detail elsewhere [6,7,16,55,65].

In view of the dynamic interactivity of virtually all mechanisms in the body [7,14,15,16,17], there is inevitably considerable interaction among the specific body functions or states, the impacts of external circumstances and the related affective experiences identified via the Model. Accordingly, factors considered within different domains may overlap; for example, a painful event may be identified in Domains 2 and 3. However, when conducting a Model-based welfare assessment, the particular origin of a specific affect needs to be considered only once, so that it should be arbitrarily assigned to a single domain. This avoids concerns about duplication that may lead to over-weighting of a particular experience in the final interpretation, and it also avoids fruitless arguments about domain specificity.

The 2015 Model [6], in common with the 1994, 2001, 2004 and 2009 versions [1,2,3,4], is generic rather than species-specific. The primary purpose of the domains is to provide examples of some internal states or external circumstances that animals may encounter and the aligned negative and positive affects that may arise in many species. However, as particular affects generated by sensory modalities that are beyond direct human experience are not known, the details provided are neither definitive nor exhaustive. Examples include unique modalities such as echolocation, ultrasonic communication, infrared sensory abilities, electromagnetic field detection, highly adapted chemical and vibrational sensitivity, as well as the exaggerated or diminished acuity of the common modalities of vision, audition and olfaction across different taxa (for References see [87]), and also, the affective experience of flight in birds, bats and gliders. Moreover, essential information about some affects and their generation is very limited or non-existent in less well-studied animals, such as in many zoo or free-living wildlife species (e.g., References [70,72,84]). For example, it is not clear whether cartilaginous fish experience some kinds of pain because of the failure, as yet, to identify the necessary sensory receptors [88]. Accordingly, each example should be assessed by reference to what is known about the animals’ species-specific behaviour, physiology and ecology considered in relation to its particular physical, biological and social environment [85].

**Figure 2:**
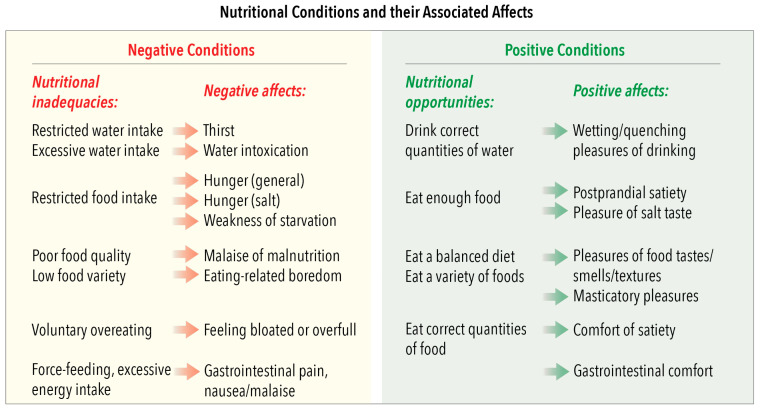
Domain 1: Nutrition. Examples of nutritional imbalances and opportunities and their associated negative and positive affects assigned to Domain 5: Mental State.

**Figure 3:**
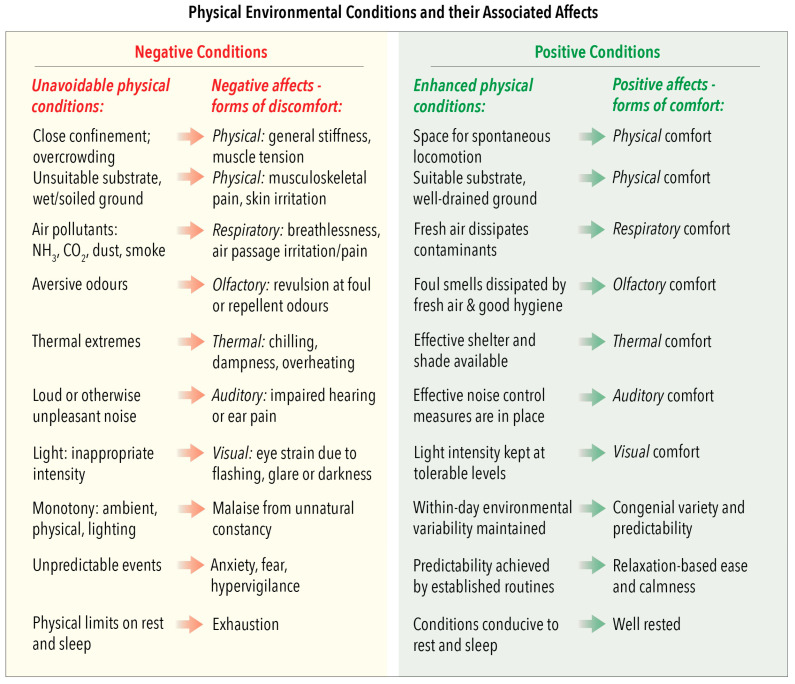
Domain 2: Physical Environment. Examples of unavoidable and enhanced physical conditions and their associated negative and positive affects assigned to Domain 5: Mental State.

**Figure 4:**
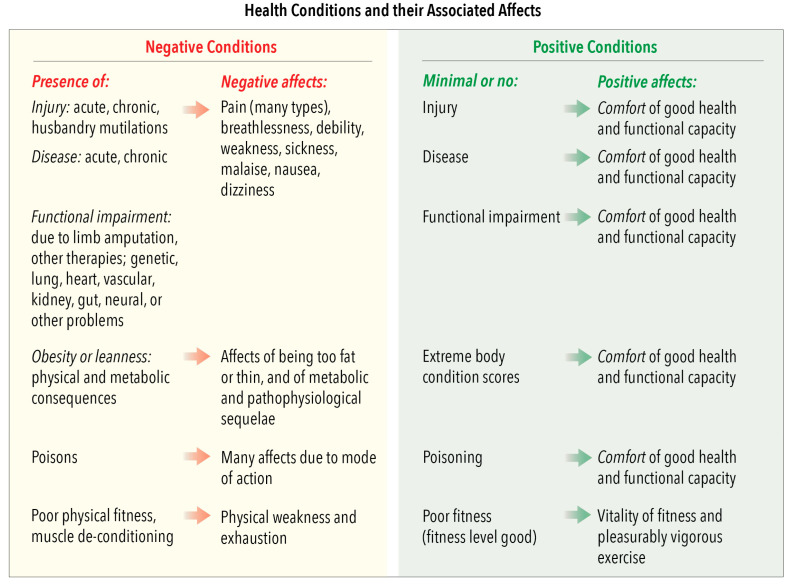
Domain 3: Health. Examples of negative and positive health conditions and their corresponding affects assigned to Domain 5: Mental State.

**Figure 5:**
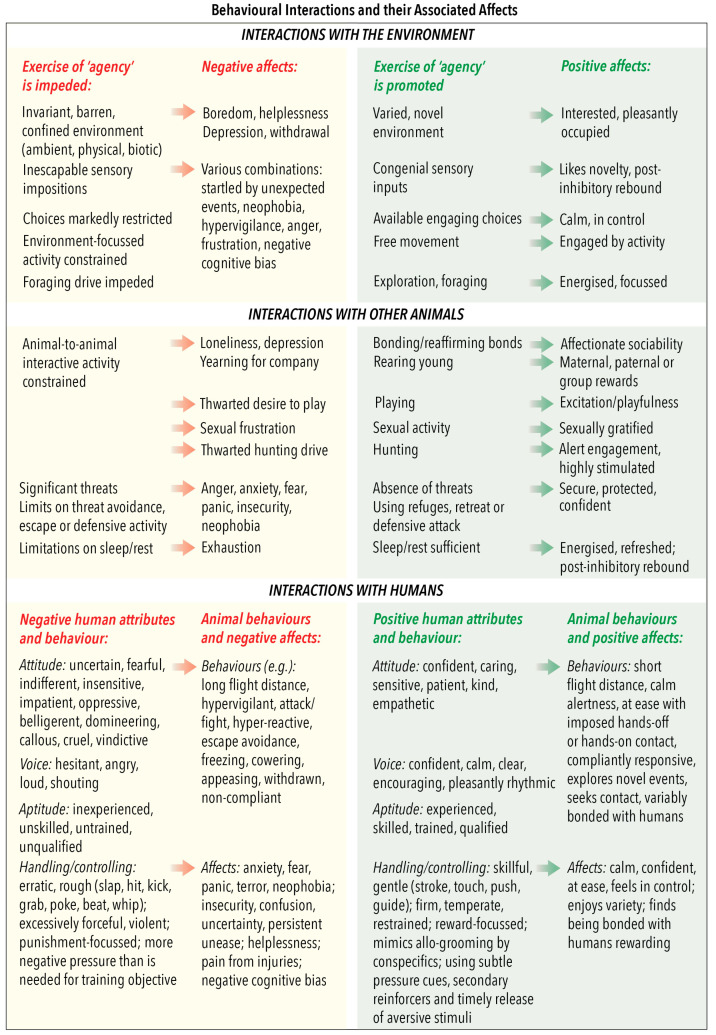
Domain 4: Behavioural Interactions. Examples of interactions with the environment, other (non-human) animals and humans, where animals’ capability to freely exercise agency would be impeded or enhanced, and examples of the corresponding affects assigned to Domain 5: Mental State. Also provided for human–animal interactions are examples of negative and positive attributes which influence the behaviour of humans towards animals.

The summary diagrams of the 2015 Model [6,7], all features of which have been included in the updated 2020 versions presented in Section 4 and Section 5 (see Figure 2, Figure 3, Figure 4 and Figure 5), have the status of guiding aides-mémoire. Therefore, when applying the Model to new species or contexts, the examples provided should be considered carefully and, only after sufficient justification, be retained, deleted or amended, and/or others added as deemed appropriate for each species (e.g., References [65,67,69,76,77,78,80,84]).

Finally, inclusion of environmental events or conditions in Domains 1 to 4 that *may* cause internally or externally derived imbalances or disruptions to the animals represent areas of increased *risk*, in which particular negative affects and welfare problems *may* arise [72,76]. However, their mere existence does not necessarily mean that the anticipated welfare problems will arise or have arisen in the particular situation under investigation. For example, the presence of potentially damaging structures in an animal’s environment presents a risk of tissue injury but does not indicate that the animal is currently experiencing welfare compromise due to pain. Any assumption of the occurrence of negative affects must be supported by directly observed animal-based physical, physiological, clinical and/or behavioural evidence [39,76]. This is equally the case for the presence of *opportunities* for animals to engage in rewarding behaviours. Clearly, there must be evidence, usually behavioural, that any such opportunities are actually *used* before their potential welfare enhancing impacts could be considered. Only then can inferences be made about any aligned negative or positive affects. This emphasises the general point that objective animal-based evidence (Domains 1 to 4) must form the foundations of any inferences about welfare-relevant affects (Domain 5) [6,7].

### 3.2. Summary of the Grading Methodology of the 2015 Model

Grading systems have been incorporated into the Model from its original formulation [1,4,6,10,65]. The bases for grading negative and positive welfare impacts differ. The defining point of reference for welfare compromise is suffering and its mitigation, whereas the focus for welfare enhancement is on animals’ use of opportunities to experience positive affective engagement [6,7,59]. The corresponding welfare impact scales also differ.

A five-tier scale (A to E) is used to grade negative welfare impacts according to the presence, intensity and/or duration of specific negative affects. Thus, grades A and B represent no and tolerably low-level impacts respectively, grade E represents very severe negative impacts related to experienced affects variously manifesting at high to very-high intensities and/or for long to very-long durations and grades C and D represent intermediate-level impacts related to their intensities and/or durations. These grades therefore equate to different degrees of welfare compromise, ranging from none to very severe [4].

Although a five-tier scale is notionally available, this does not mean that in all cases grading can be achieved with the degree of precision implied by that number of tiers. For example, when information is limited or contradictory, it may be possible to distinguish only between no to low, moderate and severe negative impacts, or, at its simplest, when a particular impact is either absent or present [6,80,81,82,89]. From the outset, numerical grading was explicitly rejected to emphasise the importance of using scientifically informed judgement, and to avoid implying, unrealistically, that much greater precision is achievable than is actually possible with such qualitative assessments [1,7,10].

In contrast, a four-tier scale (0, +, ++, +++) modified from that developed by Edgar and colleagues for poultry [58], is used to grade positive impacts where the tiers represent no, low-level, medium-level and high-level enhancement, respectively. This scale has three integrated components [6]: (1) assessment of the availability of opportunities for animals to engage in self-motivated rewarding behaviours, (2) assessment of their actual use of those opportunities and finally, (3) making cautious judgements about the degrees of positive affective engagement the animals may experience, and grading them accordingly.

Examples of grading using these two scales applied to Domain 4 are provided in Section 5.

### 3.3. The Utility of the 2015 Model for Assessing Animal Welfare

The utility of the Model, summarised here, has been evaluated in detail elsewhere [7,81]. The Model’s utility is based on validated scientific foundations of the physical/functional and behavioural indices of negative affects aligned with welfare compromise and positive affects aligned with welfare enhancement. The wide range of affects identified for consideration and the configuration of the domains that was designed specifically to clarify the likely sources of those affects, together enable Model-based welfare assessments to be structured, systematic, comprehensive and coherent. Moreover, seven interacting applications of the Model enable assessors to: (1) specify key general foci for animal welfare management, (2) highlight the foundations of specific welfare management objectives, (3) enable monitoring of responses to specific welfare-focused remedial interventions and/or maintenance activities, (4) identify previously unrecognised features of poor and good welfare, (5) facilitate qualitative grading of specific features of welfare compromise and/or enhancement, (6) enable both prospective and retrospective animal welfare assessments to be conducted and (7) provide adjunct information to support consideration of quality of life (QoL) evaluations in the context of end-of-life decisions. Nevertheless, it is important not to overstate what utilisation of the Model can achieve. Constraints arise through the following factors: (1) different levels of confidence with which particular affects may be inferred to be present in different circumstances, (2) the necessary focus only on the specific affects that can be identified, (3) differing precision with which each affect may be graded and (4) the limits imposed by an inability to determine the relative impacts of different affects when evaluating the notional overall negative–positive affective balance represented by QoL, thereby precluding the possibility of elaborating an all-inclusive QoL metric.

## 4. The 2020 Five Domains Model: Domains 1, 2 and 3

As outlined above (Section 2.3), Domains 1 to 3 direct attention towards nutritional-, environmental- and health-related survival-critical factors that disrupt or disturb discrete features of the inner stability of the body. Each form of instability has distinctive characteristics that may be identified using measurable physiological, pathophysiological, pathological, clinical and other such indices. Functionally, these indices are detected by specific sensory receptors that send neural impulses to the brain for processing into particular negative affects. Each such negative affect, generated by genetically embedded mechanisms, provides a compelling drive or motivation for the animals to engage in specific behaviours upon which their survival depends (see Section 2.3).

Although the animals would be cognitively aware of each affective experience and the aligned behaviours, they would have little or no ability to stop the behaviours from occurring. For example, the elicitation of behavioural responses to intense breathlessness, pain, nausea and dizziness would likely be almost entirely beyond an animal’s control. In contrast, some elements of choice may attend behavioural responses to other affects where the animal needs to identify and/or access locations to undertake a required corrective activity or forms of corrective inactivity. Such corrective activities include seeking water in response to thirst and locating food in response to hunger. On the other hand, corrective inactivity would likely include the seeking of restful isolation in response to debility, weakness and/or sickness. Generally, therefore, agency (i.e., animals’ ability to consciously engage in goal-directed behaviours) is not a major part of most behavioural responses to factors noted in Domains 1 to 3. In contrast, agency dominates the behavioural responses considered in Domain 4 (see Section 5).

The brief descriptions of these domains in the 2020 Model provided in Section 4.1, Section 4.2 and Section 4.3 include updated or additional examples. Note also the name change of Domain 2 from “Environment” to “Physical Environment”. This emphasises that Domain 2 directs attention towards the affective impacts of the largely physical/atmospheric conditions that animals cannot control, and to which they mount or attempt to mount obligatory physiological and pathophysiological responses, often accompanied by supportive behaviours (Section 4.2).

### 4.1. Domain 1: Nutrition—Imbalances and Opportunities and Their Associated Domain 5 Affects

This domain refers to the water and food available to animals (Figure 2). Intakes may be restricted in animals living outdoors. Examples include the following: when drought depletes natural water sources and limits the available vegetative forage or prey for hunting, when winter temperatures inhibit the growth of vegetation, when deforestation disrupts natural ecosystems, or when uncontrolled reproduction and/or overstocking raise animal numbers above the carrying capacity of rangeland or fenced areas. Poor food quality mainly refers to deficiencies or excesses of trace elements or other essential nutrients and/or inadequate energy and protein contents of plants; for example, those resulting from trace element deficiencies in soils and/or the seasonal growth cycle of grasses, or from the routine feeding of inappropriate diets, such as giving some processed dog food to cats. Low food variety refers to when animals that normally eat varied diets are given the same, albeit nutritious, foods for long periods. Examples include restricting grazing livestock to fenced areas of grass monocultures, long-term feeding of single batches of silage to dairy cows, continuously feeding a dry, nutrient-balanced processed diet to companion dogs, cats or birds and similar continuous feeding of such processed diets to laboratory animals. Negative affects elicited by these inadequacies reflect the nature of the associated welfare compromises (Figure 2).

Such compromises may be avoided or reversed when animals use nutritional opportunities that elicit the positive affects listed in Figure 2. Consideration of such nutritional problems and potential remedial actions are particularly relevant to animals maintained in enclosures that lack the full complement of nutritional conditions for which their species has evolved. This is because practically meeting animals’ nutritional requirements in ways that may elicit additional positive affects is the responsibility of the persons charged with their care. In such circumstances, the animals cannot take the required remedial actions themselves.

### 4.2. Domain 2: Physical Environment—Unavoidable and Enhanced Conditions and Their Associated Domain 5 Affects

This domain focusses attention on the affective impacts of physical and atmospheric conditions to which animals are exposed directly. When the associated affects are negative, the circumstances are categorised as unavoidable physical conditions (Figure 3) because the animals cannot escape from them. For example, in unsuitable indoor housing, these conditions may include space-, floor substrate-, atmospheric-, odorous-, thermal-, noise- and light-related factors, some of which may also lack natural variation. Each such condition is aversive and may elicit identifiable forms of discomfort. Many such conditions may also apply to animals kept outdoors, especially those maintained at high densities or confined in small enclosures; also, those unable to access shelter in cold/wet/windy conditions or shade when hot.

Remedies intended to enhance these ambient conditions can improve the animals’ welfare states by enabling them to experience various forms of comfort that may be physical, respiratory, olfactory, thermal, auditory, visual and/or variety-related (Figure 3). Attendant affective experiences may merely be neutral, because specific discomforts are absent. However, this could arguably have permissive effects by minimising unpleasant sensory inputs that would hinder the animals’ enjoyment of other experiences (see Reference [7]), for example, noxious odours obscuring attractive smells of food. Attendant affects may also be positive, for example, the pleasurable restfulness of lying on dry, soft, draught-free and hygienic substrates indoors, and the comforting thermal pleasure of basking in the sun.

### 4.3. Domain 3: Health—Negative and Positive Conditions and Their Associated Domain 5 Affects

This domain focusses attention on the welfare impacts of injury, disease and different levels of physical fitness. Injuries, whether they are acute or chronic, or caused by accidents, invasive husbandry practices, training implements, restrictive devices used to enhance performance, therapeutic surgical procedures, disease-related pathology or poisons, may cause pain that, because of its many different causes, has up to 30 different affective qualities [23]. Acute, chronic or genetic disorders, and persistent functional impairment when spontaneous or assisted recovery is incomplete, may give rise to a range of other negative affects. The character of these affects depends on the organ systems affected and the disease agent, poison and/or pathophysiological processes involved (Figure 4). Extreme overfeeding and underfeeding are included in this domain, and not Domain 1, because the associated pathophysiology may give rise to several of the negative affective experiences noted in Figure 4. Finally, fitness level is included because muscle de-conditioning and bone depletion increase susceptibility to injury and fatigue, the risks of which can be mitigated by levels of exercise that maintain muscle and bone strength (e.g., References [90,91,92]).

Achieving or maintaining good health and fitness accompanied by a wide range of positive affective experiences (Figure 4) involves using welfare-relevant husbandry practices (Domain 1), facilities design and environmental management (Domain 2) and veterinary attention (Domain 3). It also involves genetic selection for appropriate phenotypes to correct or avoid well-known functional impairments that have dire welfare consequences for production, companion and laboratory animals, and, as recently anticipated, for pest animals [93,94,95,96]. These and the previous observations in this section highlight two points: first, that factors included in the first three domains overlap due to the highly integrated functional interactivity within the body operating as an integrated entity (Section 2.1), and second, that these three domains deal mainly with survival-critical conditions and their associated affects (Section 2.3).

## 5. The 2020 Five Domains Model: Including Human–Animal Interactions in Domain 4

Domain 4, previously named “Behaviour” (Figure 1), has been renamed “Behavioural Interactions” (Figure 5) in order to give greater clarity to its role in the Model. Whereas Domains 1 to 3 mainly focus on animal care-related inputs to welfare, Domain 4 is intended to capture behavioural outputs as indices of animals’ perceptions of their external circumstances. More specifically, it highlights the flexible agency-related behaviours animals mount in response to variable, often unpredictable external events and conditions.

Agency is apparent when animals engage in voluntary, self-generated and/or goal-directed behaviours [42,49,53]. More specifically, agency indicates the intrinsic propensity (genetic and/or learned) of an animal to actively engage with its physical, biological and social environment, beyond the degree demanded by its momentary needs, in order to gather knowledge and enhance its skills for future use in responding effectively to varied and novel challenges [42,49,53]. In other words, the exercise of agency involves the cognitive assessment of circumstances in support of animals making mainly conscious choices to behave in particular ways [97,98].

Accordingly, the primary focus of Domain 4 is on behavioural evidence of hindered and/or enhanced expression of agency when animals interact with (1) their environment, (2) other non-human animals and (3) human beings. For these interactions, the aligned affects are largely produced by brain processing of sensory inputs elicited from outside the body. Hence, Domain 4 captures agency-focussed responses to situation-related factors. As noted above (Section 4), this contrasts with the focus of the first three domains on genetically programmed physiological and/or pathophysiological mechanisms inside the body that are specifically directed towards restoring and/or maintaining survival-enhancing internal stability [6,7].

Although the 2015 Model included interactions with the environment and other non-human animals, reference to them was not differentiated structurally. In addition, as mentioned above, human–animal interactions were not included specifically, but were noted as meriting consideration [6]. All three categories are identified explicitly in the 2020 Model.

### 5.1. Features Common to All Three Categories of Behavioural Interaction

Operationally, the three categories focus on behaviour-based evaluations of affective experiences that animals may have when they direct their attention externally (Figure 5). In terms of impediments, particular negative affects are anticipated when specific agency-related behaviours are absent, or their occurrence is diminished in animals occupying severely restricted, oppressive and/or challenging circumstances, such as those noted in Figure 5. The generation of these affects is considered to result, at least partly, from thwarting of genetically programmed elements of an animal’s ethogram, by disabling its engagement in rewarding behaviours and/or by a failure to gain anticipated rewards [44,49,99]. Examples include the following: (1) the daily thwarting of normal long-duration grazing motivation in stabled horses fed with highly concentrated feeds which nevertheless meet their nutritional requirements, (2) the frustrated hunting motivation of canids and felids kept indoors with no suitable substitutes, (3) the frustration of social species such as horses and elephants that are prevented from joining conspecifics engaged in social behaviours, (4) the yearning for company (i.e., loneliness) of isolated individuals of social species kept in separate enclosures and (5) the “separation anxiety” in strongly bonded companion animals due to withdrawal of human company and physical contact.

The opposite of thwarted motivation arises in circumstances that provide opportunities which enhance animals’ ability to express agency-related behaviours (Figure 5). Providing such opportunities allows situation-related negative affects to be replaced by positive affects, thereby enabling animals to experience states of “positive affective engagement” [6,7,16,59,60]. Such engagement represents the experience animals may have when they respond to motivations to undertake behaviours that they find rewarding, and it potentially incorporates all of the associated affects that are positive [59,60]. More specifically, enhanced circumstances enable animals to respond to genetically programmed or learned impulses to engage in agency-related behaviours that are linked to affectively positive experiences of anticipation, goal achievement and memory of success [35,37,45,51,53,59,60,63]. Moreover, as the exercise of agency is anticipated to be accompanied by animals having a general sense of being in control of their actions [49,53,63], this would further enhance their feelings of mental security and experiences of positive affective engagement [59,63].

Finally, positive experiences may also arise in ways not directly related to the exercise of agency [6,12]. Examples include the following: (1) herbivores enjoying the pleasant tastes and textures of a variety of feeds delivered to them indoors, just as they would when they self-select and ingest the same feeds while grazing outdoors, (2) the companionable benefits enjoyed by bonded animals yarded together, duplicating those requiring them to actively locate and maintain contact with each other when part of groups on open ranges and (3) humans initiating and maintaining interactive contact with companion animals in the home in ways that provide satisfaction which approximates to that of agency-instigated affiliative interactions between conspecifics in pre-domestication circumstances.

### 5.2. Animals’ Interactions with Humans

The principal focus of human–animal interactions is the impacts of the presence and behaviour of persons as primary causes of animals’ behavioural and affective responses. This emphasis includes both animal training and husbandry. In the trained animal, examples are the effects on agency of altered cues and contingencies of learned responses such as ambiguous signals, relentless tactile pressures and altered expectations of reward [100]. Underscoring this, it is well established that: (1) the attitudes, motivation, understanding and skills training of people influence the nature of their behaviour towards animals, (2) it is the impact of their behaviour on the animals that elicits animals’ negative and/or positive affective experiences and (3) the nature of the animals’ experiences may be inferred from their behavioural and physiological responses (e.g., References [12,29,55,60,87,101,102,103,104,105,106,107,108,109,110,111,112,113,114,115]).

Figure 5 provides examples of salient human characteristics, subdivided according to attitude, voice, aptitude and handling/control, as well as examples of animals’ impeded or enhanced agency-related behaviours and their aligned affective experiences. The examples are just that. They are neither definitive nor exhaustive, nor should they be generalised to all animals. Also, the listed negative and positive human attributes and animals’ affective experiences are intended to indicate possible negative-to-positive ranges, thereby facilitating consideration of these factors at and between these extremes. As with all other animal-based examples provided in Figure 5 (also in Figure 2, Figure 3 and Figure 4), users of the Model should evaluate them with regard to any unique behavioural, biological and ecological features of the species in question, together with the precise circumstances of the animals being considered. The purpose here is for Model users to decide whether any named human behaviours and/or induced animal behaviours or affects should be deleted, retained or modified, or whether others should be added.

**Figure 6:**
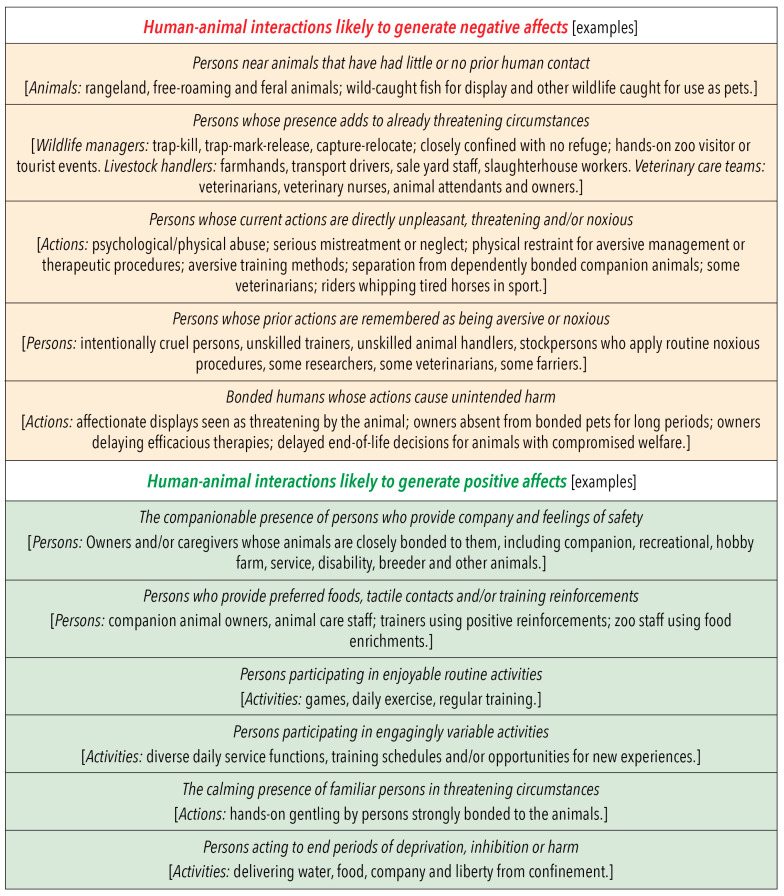
Some general circumstances in which the presence of humans at a distance, close to or in direct contact with animals may lead the animals to have negative or positive affective experiences, and some specific examples of those circumstances. The examples provide an indication of the negative-to-positive range of human–animal interactions. These, when considered together with the negative-to-positive range of influential human attributes illustrated in Figure 5, are provided to help Model users to evaluate in more detail the impacts of interactions at and between these extremes.

As an adjunct to Figure 5, Figure 6 lists some of the general circumstances in which Model use could include assessments of the impacts on animals of specific negative and/or positive features of human proximity and/or behaviour. It also provides examples of activities or occupations where those circumstances may apply.

More than one of these general circumstances may be applicable to particular examples of human–animal interactions if they develop over time or when different interactions occur in sequence. Also, the examples in Figure 6 are not exhaustive. Rather, their purpose, as with the examples in Figure 2, Figure 3, Figure 4 and Figure 5, is to highlight a range of factors that the Model may be used to evaluate. We encourage the introduction of other examples that may be more applicable to the circumstances and the species-specific attributes of the animals being considered.

### 5.3. Grading the Negative and Positive Impacts of Humans in Their Interactions with Animals

The grading of welfare impacts in Domain 4 is focussed on the observable behaviour of animals during and following their interactive engagement with (1) different features of their environment, (2) other non-human animals and (3) humans in their vicinity (Section 5.2). Of course, such impact grading must also include any germane elements of the wider circumstances of the animals as revealed by all other aspects of the Model assessment, captured via Domains 1 to 3 (Section 4.1, Section 4.2 and Section 4.3).

**Figure 7:**
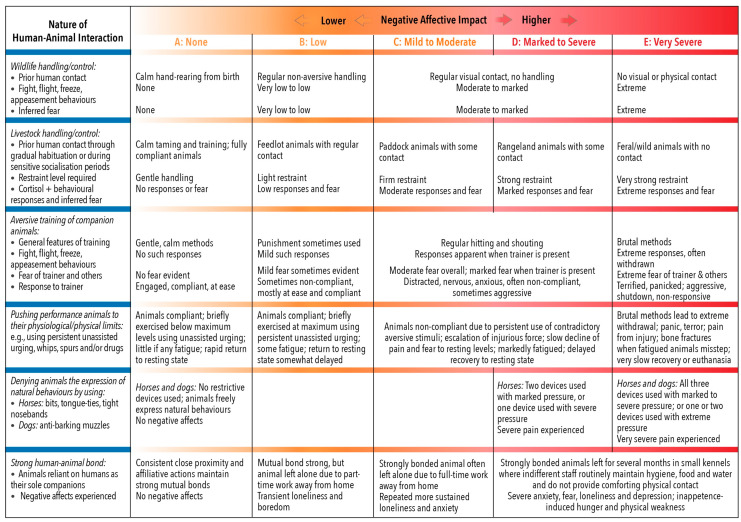
Examples of graded negative affective impacts due to different human interactions with animals of different types and in different situations. For each type of interaction, grades indicated in each row relate to variations in relevant factors of the interaction, such as the animal’s prior contact with humans or the training regime. Also noted for each sub-scenario is the degree to which behavioural and/or physiological indicators of the affective experience are expressed by the animal, as well as the intensity of specific inferred negative affects, e.g., fear. The approach here is therefore similar to the grading of other negative impacts.

**Figure 8:**
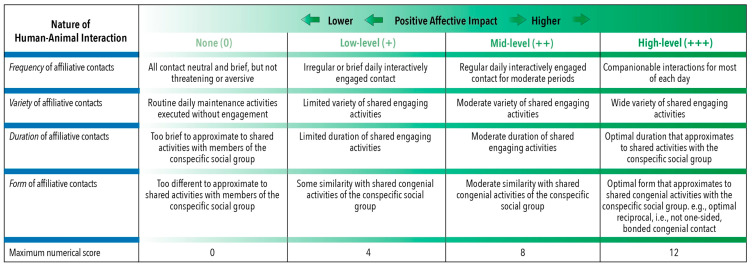
Examples of relative positive affective impacts on animals due to human interactions graded separately according to the frequency, variety, duration and form of congenial contacts. As these four features interact, they all need to be graded for each situation, and their grades amalgamated into an overall grade. For this purpose, a numerical score is applied to each feature in each column. Note that this is a numerical aid to the qualitative assessment of positive impacts. If any feature receives a zero score, none of the other levels apply and the overall score is zero. The minimum overall score above zero is 4 (1 for each feature), an intermediate score is 8 (2 for each feature) and the maximum is 12 (3 for each feature). The range of possible overall scores above zero in each situation would therefore be 4 to 12. As each feature is graded qualitatively before amalgamation, each overall numerical score is merely a guide for prospectively or retrospectively comparing outcomes of proposed or completed changes by undertaking a succession of such assessments. Note that such comparisons within specific situations is qualitatively meaningful, whereas such comparisons between different situations is not.

More specifically, the grading of the impacts of the proximity and animal-centred behaviour of humans employs the same two scales as for all other features of Domain 4 (Figure 5), as also of Domains 1 to 3 (Figure 2, Figure 3 and Figure 4). Hence, it uses the five-tier scale (A to E) for negative impacts and the four-tier scale (0, +, ++, +++) for positive impacts (Section 3.2). However, the examples of graded negative impacts in Figure 7 and graded positive impacts in Figure 8 highlight an important difference.

The grading of negative impacts is based on assessments of the physiological, behavioural and clinical impacts of human proximity and behaviour on the animals. Thus, the grades in each row of Figure 7 represent a separate relative assessment of variants of particular situations, for example, behavioural responses of wildlife that have had different levels of prior exposure to humans.

In contrast, illustrated in Figure 8 is the grading of the positive impacts of affiliative human–animal interactions, where four key features of human-initiated interactions are emphasised; namely, frequency, variety, duration and form. As these four features also interact, they all need to be graded for each situation, and their grades amalgamated into an overall grade (see Figure 8 for more details). What is presented, therefore, is a means of more thoroughly assessing the human contributions to positive impacts in a wide range of situations. As these assessments are qualitative, the overall grades for different situations cannot be compared meaningfully, but repeated assessments of the same system to detect negative or positive changes would be meaningful (Figure 8).

Although devised here for the assessment of positive human-animal interactions, reference to the four key interactive features of frequency, variety, duration and form of interaction noted above also has application to assessments of positive animal-to-environment and animal-to-animal interactions. We anticipate the need to explore the influences, relevance and consequences of various behavioural conditioning techniques (i.e., training) on the welfare of animals, as viewed through the Five Domains lens. In particular, there will be value in assessing interactions between the outcomes of different modes of learning (associative and non-associative), considered against the backdrop of the animals’ evolved capacities to function and behave in species-specific ways, i.e., in relation to their telos (see References [116,117]).

## 6. Conclusions

Renaming Domain 4 “Behavioural Interactions” (previously “Behaviour”), highlights the inherent capability of sentient animals to consciously self-select goal-directed behaviours when interacting with key features of their environment, with other non-human animals and with humans. When they achieve their selected goals, they may experience one or more of a wide range of welfare-enhancing positive affects (Figure 5). These are rewarding and provide motivation to again engage in the selected behaviours, subjectively experienced as different forms of ‘positive affective engagement’ [59]. In contrast, if the external circumstances hinder animals from engaging in behaviours that they would find rewarding, they may experience one or more of a range of unpleasant and demotivating negative affects (Figure 5) [6,7]. Animals’ agency-related interactions with their environment and with other non-human animals in their environment have been described in detail previously (for References see [6,7,12,16,21,51,59,60]). However, humans also feature as influential in animals’ external circumstances, and their interactive behaviour towards animals has the potential to elicit welfare-enhancing positive affects or welfare-compromising negative affects. The Five Domains Model as reconfigured here now provides an explicit means to effectively and systematically evaluate the animal welfare implications of a wide range of human–animal interactions. This extension of the Model is therefore recommended to readers for their consideration and use.

To assist users of the 2020 Model, we have prepared a freely available online summary poster, which combines Figure 2, Figure 3, Figure 4 and Figure 5 [118].
